# Attention-based multi-semantic dynamical graph convolutional network for eeg-based fatigue detection

**DOI:** 10.3389/fnins.2023.1275065

**Published:** 2023-11-21

**Authors:** Haojie Liu, Quan Liu, Mincheng Cai, Kun Chen, Li Ma, Wei Meng, Zude Zhou, Qingsong Ai

**Affiliations:** School of Information Engineering, Wuhan University of Technology, Wuhan, Hubei, China

**Keywords:** EEG, driving fatigue detection, channel attention mechanism, graph convolutional network, spatial attention mechanism

## Abstract

**Introduction:**

Establishing a driving fatigue monitoring system is of utmost importance as severe fatigue may lead to unimaginable consequences. Fatigue detection methods based on physiological information have the advantages of reliable and accurate. Among various physiological signals, EEG signals are considered to be the most direct and promising ones. However, most traditional methods overlook the functional connectivity of the brain and fail to meet real-time requirements.

**Methods:**

To this end, we propose a novel detection model called Attention-Based Multi-Semantic Dynamical Graph Convolutional Network (AMD-GCN). AMD-GCN consists of a channel attention mechanism based on average pooling and max pooling (AM-CAM), a multi-semantic dynamical graph convolution (MD-GC), and a spatial attention mechanism based on average pooling and max pooling (AM-SAM). AM-CAM allocates weights to the input features, helping the model focus on the important information relevant to fatigue detection. MD-GC can construct intrinsic topological graphs under multi-semantic patterns, allowing GCN to better capture the dependency between physically connected or non-physically connected nodes. AM-SAM can remove redundant spatial node information from the output of MD-GC, thereby reducing interference in fatigue detection. Moreover, we concatenate the DE features extracted from 5 frequency bands and 25 frequency bands as the input of AMD-GCN.

**Results:**

Finally, we conduct experiments on the public dataset SEED-VIG, and the accuracy of AMD-GCN model reached 89.94%, surpassing existing algorithms.

**Discussion:**

The findings indicate that our proposed strategy performs more effectively for EEG-based driving fatigue detection.

## 1 Introduction

Drivers driving for a long time or driving at night can lead to a decline in physical and psychological abilities, seriously affecting the ability to drive safely. Fatigue while driving can impair basic skills such as attention, decision-making, and reaction time, while also affecting cognitive processes, sensory perception, and overall mental well-being. In severe cases, this may result in a decline in motor function and increase the likelihood of being involved in traffic accidents. Statistically, in 2004, the World Health Organization released the “World Report on Road Traffic Injury Prevention”, which pointed out that approximately 20%~30% of traffic accidents were caused by fatigue driving. By 2030, the number of road traffic fatalities is projected to rise to about 2.4 million people annually, making road traffic deaths the fifth leading cause of death worldwide (WHO, 2009). As the number of casualties due to fatigue driving continues to increase, it is urgent to develop reliable and effective driving fatigue detection methods.

The existing fatigue detection methods mainly include vehicle information-based, facial feature-based, and physiological signal-based approaches. The vehicle information-based detection method indirectly assess the driver's fatigue state based on the driver's manipulation of the vehicle (Li et al., 2017; Chen et al., 2020). This method utilizes on-board sensors and cameras to collect data such as steering wheel angle, grip force, vehicle speed, and driving trajectory. By analyzing the differences in driving behavior parameters between normal driving and fatigue states, it assesses the driver's fatigue condition. However, it is challenging to collect accurate and stable data using this method due to variations in driving habits and proficiency among drivers. The facial feature-based detection method infers the driver's fatigue state through analyzing eye status, mouth status, and head posture (Wu and, 2019; Quddus et al., 2021; Huang et al., 2022). This method mainly uses the camera to capture the driver's face image, and extracts the fatigue-related information through the computer vision technology. In contrast, physiological signal-based detection methods can directly reflect the driver's driving state, including electroencephalogram (EEG), electrooculogram (EOG), electrocardiogram (ECG), and electromyogram (EMG). Among various physiological signals, EEG signals contain all the information of brain operation and are closely related to mental and physical activity, with good time resolution and strong anti-interference ability (Yao and Lu, 2020), which are the result of excitatory or inhibitory postsynaptic potentials generated by the cell bodies and dendrites of pyramidal neurons (Zeng et al., 2021). Meanwhile, the EEG caps tend to be intelligent and lightweight (Lin et al., 2019), making it convenient to keep an EEG cap while driving. EEG signals are considered the most direct and promising.

EEG signals are recordings of the spontaneous or stimulus-induced electrical activity generated by specific regions of the brain's neurons during physiological processes, reflecting the brain's biological activities and carrying a wealth of information (Jia et al., 2023). From an electrophysiological perspective, every subtle brain activity induces corresponding neural cell discharges, which can be recorded by specialized instruments to analyze and decode brain function. EEG decoding is the separation of task-relevant components from the EEG signals. The main method of decoding is to describe task-related components using feature vectors, and then use classification algorithms to classify the relevant features of different tasks. The accuracy of decoding depends on how well the feature algorithm represents the relevant tasks and the discriminative precision of the classification algorithm for different tasks. The EEG signals record the electrical wave changes in brain activity, making them the most direct and effective reflection of fatigue state. Based on the amplitude and frequency of the waveforms, EEG waves are classified into five types: δ(1-3Hz), θ(4-7Hz), α(8–13Hz), β(14–30Hz), γ(31–50Hz) waves (Song et al., 2020). It is worth noting that, during the awake state, EEG signals are mainly characterized by α and β waves. As fatigue increases, the amplitude of α and β waves gradually diminishes, and they may even disappear, while δ and θ waves gradually increase, indicating significant variations in EEG signals during different stages of fatigue (Jia et al., 2023). Therefore, many scholars regard EEG signals as the gold standard for measuring the level of fatigue (Zhang et al., 2022). Lal and Craig (2001) tested non-drivers' EEG waves and analyzed the characteristics of EEG wave changes in five stages: non-fatigue, near-fatigue, moderate fatigue, drowsiness, and anti-fatigue. They concluded that EEG is the most suitable signal for evaluating fatigue. Lal and Craig (2002) collected EEG data from 35 participants in the early stage of fatigue using 19 electrodes. The experimental results indicated a decrease in the activity of α and β waves during the fatigue process, while there was a significant increase in the activity of δ and θ waves. Papadelis et al. (2006) introduced the concept of entropy in a driving fatigue experiment. The study found that under severe fatigue conditions, the number of α waves and β waves exhibited inconsistent changes, and shannon entropy and kullback-leibler entropy values decreased with the changes in β waves.

In recent years, thanks to the rapid development of sensor technology, information processing, computer science, and artificial intelligence, a large number of studies have proposed combining fatigue driving detection based on EEG signals with machine learning or deep learning methods. Paulo et al. (2021) proposed using recursive graphs and gramian angular fields to transform the raw EEG signals into image-like data, which is then input into a single-layer convolutional neural network (CNN) to achieve fatigue detection. Abidi et al. (2022) processed the raw EEG signals using a tunable Q-factor wavelet transform and extracted signal features using kernel principal component analysis (KPCA). They then used k-nearest neighbors (KNN) and support vector machine (SVM) for EEG signal classification. Song et al. (2022) proposed a method that combines convolutional neural network (CNN) and long short-term memory (LSTM) called LSDD-EEGNet. It utilizes CNN to extract fe atures and LSTM for classification. Gao et al. (2019) introduced core blocks and dense layers into CNN to extract and fuse spatial features, achieving detection. In the study (Wu et al., 2021), designed a finite impulse response (FIR) filter with chebyshev approximation to obtain four EEG frequency bands (i.e., δ, θ, α, β), and constructed a new deep sparse contracting autoencoder network to learn more local fatigue features. Cai et al. (2020) introduced a new method referred to as graph-time fusion dual-input convolutional neural network. This method transforms each EEG epoch of sleep stages into limited penetration visible graph (LPVG) and utilizes a new dual-input CNN to assess the degree sequences of LPVG and the original EEG epochs. Finally, based on the CNN analysis, the sleep stages are classified into six states. Gao et al. (2021) were the first to explore the application of complex networks and deep learning in EEG signal analysis. They introduced a fatigue driving detection network framework that combines complex networks and deep learning. The network first calculates the EEG signals for each channel and generates a feature matrix using a recursive rate. Then, this feature matrix is fed into a specially designed CNN, and the prediction results are obtained through the softmax function.

The above deep learning and convolutional neural network (CNN) methods mainly focus on the features of individual electrode EEG signals and overlook the functional connectivity of the brain, that is the correlation between EEG channels. Due to the non-Euclidean structure of EEG signals, CNN based on Euclidean space learning is limited in handling the functional connections between different electrodes. Therefore, using CNN to process EEG signals may not be an optimal choice.

In recent years, the emergence of graph convolutional neural networks (GCN) has been proven to be the most effective method for handling non-Euclidean structured data (Jia et al., 2021; Zhu et al., 2022). Using GCN to process EEG signals allows to represent the functional connections of the brain through topological data. In this case, each EEG signal channel is treated as a node in the graph, and the connections between EEG signal channels serve as the edges of the graph. Jia et al. (2023) proposed a model called MATCN-GT for fatigue driving detection, which consists of a multi-scale attention time convolutional neural network block (MATCN) and a graph convolution-transformer (GT) block. The MATCN directly extracts features from the raw EEG signals, while the GT processes the features of EEG signals from different electrodes. Zhang et al. (2020) introduced the PDC-GCNN method for detecting driver's EEG signals, which uses partial directed coherence (PDC) to construct an adjacency matrix, and then employs graph convolutional neural network (GCN) for EEG signal classification. Song et al. (2020) proposed a multi-channel EEG emotion recognition method based on dynamic graph convolutional neural network (DGCNN). The basic idea is to use graphs to model multi-channel EEG features and then perform EEG emotion classification based on this model. Jia et al. (2020) proposed a novel deep graph neural network called GraphSleepNet to classify EEG signals. This network can dynamically learn the adjacency matrix and utilizes a spatio-temporal graph convolutional network (ST-GCN) to classify EEG signals. The method demonstrated excellent classification results on the MASS dataset. Zhang et al. (2019) designed a graph convolution broad network (GCB-net) to explore deeper-level information in graph-structured data. It utilizes graph convolutional layers to extract features from the input graph structure and stacks multiple regular convolutional layers to capture more abstract features. Additionally, a broad learning system (BLS) is employed to enhance the features and improve the performance of GCB-net.

Although GCN is proficient at learning the internal structural information of EEG signals, it relies on the connectivity between nodes provided by the adjacency matrix. Most methods obtain functional connectivity of EEG signals by using predefined fixed graphs such as PLI, PLV, PDC, or spatial relationships, which prevents the model from adaptively constructing adjacency graphs simultaneously related with subjects, fatigue states and samples, thereby overlooking the data-driven intrinsic correlations. However, constructing a suitable graph representation for the adjacency matrix of each data in advance requires time and effort. Additionally, GCN faces challenges in learning dependencies between distant nodes (long-range vertices). Increasing the depth of GCN to expand the receptive field remains difficult and may lead to over-smoothing of nodes.

To address the above problem, we propose a new fatigue driving detection network, referred to as the attention-based multi-semantic dynamical graph convolutional network (AMD-GCN). First, the network utilizes a channel attention mechanism based on average pooling and max pooling to assign weights to the fused EEG input features. This helps the model focus on the crucial information parts related to fatigue detection. Next, the adjusted EEG input features are fed into the GCN, we determine the adjacency matrix using spatial adjacency relationships, Euclidean spatial distances, and self-attention mechanism to construct data-driven intrinsic topology under multiple semantic patterns, thereby enhancing the spatial feature extraction capability of GCN. Furthermore, a spatial attention mechanism based on average pooling and max pooling is employed to calculate the weights of spatial nodes in the output of GCN, which helps in removing redundant node information and reducing interference in fatigue detection. Finally, the prediction results are output by softmax.

## 2 Dataset description and EEG pre-processing

### 2.1 Public dataset SEED-VIG

**Figure 1:**
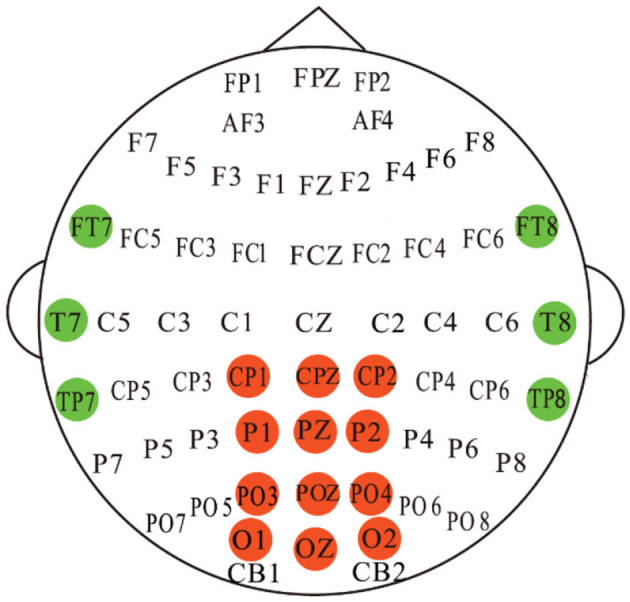
Electrode placements for the EEG setups. 12-channel and 6-channel EEG signals were recorded from the posterior region (red color) and the temporal region (green color), respectively.

We validated the proposed method on the publicly available dataset SEED-VIG (Zheng and Lu, 2017) for driving fatigue detection researches. SEED-VIG adopt the international 10-20 electrode system standard, and the EEG signals were collected from 6 channels in the temporal region of the brain (FT7, FT8, T7, T8, TP7, TP8) and 12 channels from the posterior region (CP1, CPZ, CP2, P1, PZ, P2, PO3, POZ, PO4, O1, OZ, O2), where CPZ channel serves as the reference electrode, and the specific electrode placement is shown in Figure 1. The experiment simulated a driving environment by creating a virtual reality scenario, in which 23 participants engaged in approximately 2 hours of simulated driving during either a fatigue-prone midday or evening session. The subjects comprised 12 females and 11 males, with an average age of 23.3 years and a standard deviation of 1.4. All subjects had normal or corrected vision.

The SEED-VIG dataset was vigilantly annotated using eye-tracking methods, capturing participants' eye movements with the assistance of SMI eye-tracking glasses. These glasses categorized eye states into fixation, blink, and saccade, and recorded their respective durations. The “CLOS” state, referring to slow or long-duration eye closure, is undetectable by the SMI eye-tracking glasses. In such cases, fixation and saccade represent normal states, while blink or CLOS indicates fatigue in participants. Therefore, PERCLOS represents the percentage of time in a specific period when participants were in a fatigued state (Dinges and Grace, 1998). The calculation of PERCLOS is as follows:


(1) $$\begin{array}{l} PERCLOS=\frac{blink+close}{interval},\\ interval=blink+fixation+saccade+close \end{array}$$

Where blink, close, fixation, and saccade denote the duration of eye states (blink, close, gaze, and sweep, respectively) recorded by the eye tracker within the 8-second intervals. PERCLOS is a continuous value between 0 and 1, with smaller values indicating higher vigilance. The standard procedure for using this publicly available dataset for research is to set two thresholds (0.35 and 0.7) in order to classify the samples into three types:

- Awake class: *PERCLOS* < 0.35;
- Tired class: 0.35 ≤ *PERCLOS* < 0.7;
- Drowsy class: *PERCLOS* ≥ 0.7.

In addition, we validated our proposed method on the SEED-VIG dataset, dividing each subject's 885 samples into 708 samples for training and 177 samples for testing by a way that preserves the temporal order, then we trained the model separately on each subject and evaluated it on the testing samples of the same subject. Finally, in order to mitigate the impact of data imbalance within one subject on the model performance evaluation as much as possible, the average classification accuracy and individual variation of 23 subjects were computed as evaluation metrics. It is worth noting that SEED-VIG adopts an 8-second non-overlapping sliding window to sample data, and we split the dataset by preserving the temporal order. Therefore, training is based on past data, and testing is based on future data. This ensures that the model is evaluated on unseen data, thereby alleviating the risk of data leakage (Saeb et al., 2017).

### 2.2 EEG pre-processing

The signal preprocessing method is consistent with other works (Zheng and Lu, 2017; Ko et al., 2021; Peng et al., 2023; Shi and Wang, 2023), we directly used the clean EEG signals provided by the study (Zheng and Lu, 2017), which has removed eye blinks, and the raw EEG data was downsampled from 1000 Hz to 200 Hz to reduce computational burden. Subsequently, it is bandpass filtered between 1-50 Hz to remove irrelevant components and power line interference. For SEED-VIG, there are two different methods to segment the frequency range into different bands. One widely used approach is to divide the frequency range into bands as follows: δ(1-3Hz), θ(4-7Hz), α(8-13Hz), β(14-30Hz), γ(31-50Hz). The other method is to uniformly divide the range into 25 bands with a 2-Hz resolution.

For each frequency band, the computation of the extracted differential entropy (DE) feature is as follows:


(2) $$\begin{array}{l} h\left(X\right)=-\int_{X}f\left(x\right)\ln\;f\left(x\right)dx \end{array}$$

Here, *X* is a random variable whose probability density function is defined by *f*(*x*). Assuming that the probability density function *f*(*x*) of the EEG signal follows the Gaussian distribution *N*(μ, δ^2^), the DE feature can then be computed as:


(3) $$\begin{array}{l} \begin{array}{c} h\left(X\right)=-\int f\left(x\right)\left(-\frac{1}{2}\ln\;\left(2\pi \delta^{2}\right)-\frac{{\left(x-\mu \right)}^{2}}{2\delta^{2}}\right)\\ =\frac{1}{2}\ln\;\left(2\pi \delta^{2}\right)+\frac{Var\left(X\right)}{2\delta^{2}}=\frac{1}{2}\ln\;\left(2\pi e\delta^{2}\right) \end{array} \end{array}$$

Here, we used the facts that ∫ *f*(*x*)*dx* = 1 and *Var*(*x*) = ∫ *f*(*x*)(*x* − μ)^2^*dx* = δ^2^. DE features were extracted by short-term Fourier transform with an 8-second non-overlapping time window.

The overall properties of SEED-VIG are summarized in Table 1. In our study, we concatenate the DE features extracted based on 5 frequency bands and the DE features extracted based on 25 frequency bands within the same time window as one sample input to the neural network. This allows us to fully utilize the information contained in the original EEG signal and thereby enhance the effect of fatigue driving detection. The overall data form of one subject can be expressed as *R*^885×17×30^.

**Table 1:** Summary of the overall properties of SEED-VIG.

**Dataset**	**Samples**	**Channels**	**Frequency bands**
SEED-VIG-5band	885	17	5
SEED-VIG-2Hz	885	17	25
PERCLOS-labels	885	N / A	N / A

NA, Not Applicable.

## 3 Method

**Figure 2:**
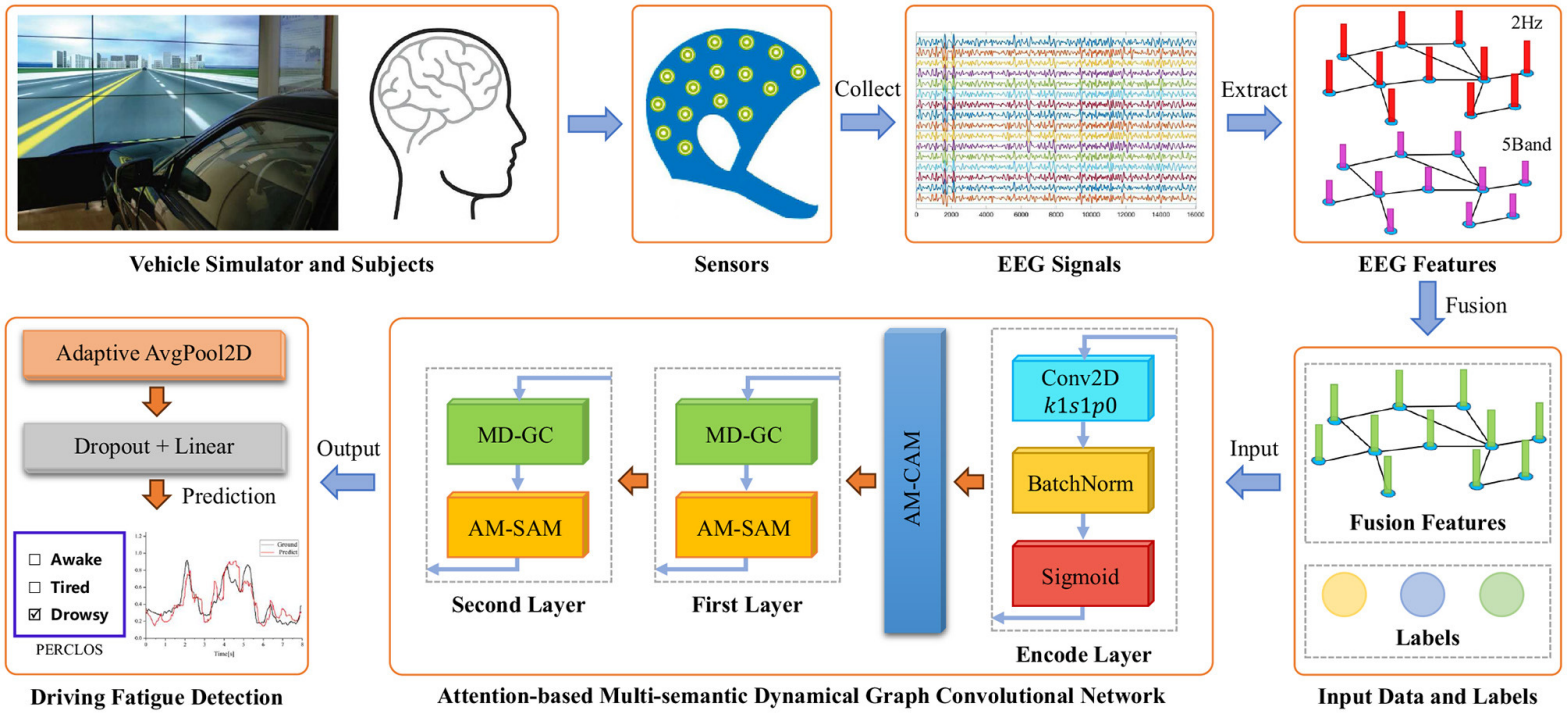
Overall schematic diagram of fatigue driving detection based on AMD-GCN. AMD-GCN consists of three modules: AM-CAM module, MD-GC module, and AM-SAM module. The input to the model is the fused feature of DE features extracted based on 5 frequency bands and DE features extracted based on 25 frequency bands. The output is the predicted label with probabilities.

Our proposed AMD-GCN model consists of three functional modules: channel attention mechanism based on average pooling and max pooling (AM-CAM), multi-semantic dynamical graph convolution (MD-GC), and spatial attention mechanism based on average pooling and Max pooling (AM-SAM). The AMD-GCN model enables end-to-end fatigue state assessment of drivers based on the extracted DE features from EEG signals. The AMD-GCN model retains crucial input features through AM-CAM, performs multi-semantic spatial feature learning through MD-GC, and eliminates redundant spatial nodes information through AM-SAM. The overall architecture of fatigue driving detection based on AMD-GCN is illustrated in Figure 2.

### 3.1 Preliminary

In our paper, we designed the AMD-GCN model adopting graph convolutional neural networks to process spatial features. To facilitate reader comprehension, we first elucidate the fundamental concepts and relevant content of GCN before introducing AMD-GCN.

Consider a graph *G* = (*V*, ε, *A*), which represents a collection of all nodes and edges. Here, *V* = (*v*_1_, *v*_2_, ..., *v*_*n*_) signifies that the graph has *N* nodes, *v*_*n*_ denotes the *n*-th node, and *E* is a set of edges representing relationships between nodes. *A* ∈ *R*^*N*×*N*^ stands for the adjacency matrix of graph G, denoting connections between two nodes. It's worth noting that GCN (Kipf and Welling, 2016) employs graph spectral theory for convolutional operations on topological graphs. It primarily explores the properties of the graph through the eigenvalues and eigenvectors of the graph's Laplacian matrix. The Laplacian matrix of a graph is defined as follows:


(4) $$\begin{array}{l} L=D-A \end{array}$$

where *D* ∈ *R*^*N*×*N*^ is the degree matrix of the vertices (diagonal matrix), that is, the elements on the diagonal are the degrees of each vertex in turn. *L* denotes the Laplacian matrix, whose normalized form can be expressed as:


(5) $$\begin{array}{l} L=I_{n}-D^{-\frac{1}{2}}AD^{-\frac{1}{2}}=UAU^{T} \end{array}$$

Where *I*_*n*_ is the identity matrix. *UAU*^*T*^ represents the orthogonal decomposition of the Laplacian matrix, where $U=\left[u_{0},u_{1},...,u_{n-1}\right]\in R^{n\times n}$ is the orthogonal matrix of eigenvectors obtained through the singular value decomposition (SVD) of the graph Laplacian matrix, and $\Lambda =\left[\lambda_{0},\lambda_{1},...,\lambda_{n-1}\right]\in R^{n\times n}$ is the diagonal matrix of corresponding eigenvalues. For a given input feature matrix *X*, its graph Fourier transform is:


(6) $$\begin{array}{l} \hat{X}=U^{T}X,X=U\hat{X}\left(inverse\right) \end{array}$$

The convolution of the graph for input *X* and filter *K* can be expressed as:


(7) $$\begin{array}{l} Y=X*GK=U\left(\left(U^{T}X\right)⊙\left(U^{T}G\right)\right)=U\hat{K}U^{T}X \end{array}$$

Here, ⊙ denotes the element-wise Hadamard product. However, directly computing the Eq.7 would require a substantial amount of computational resources. To mitigate energy consumption, Kipf and Welling (2016) proposed an efficient variant of convolutional neural networks that directly operate on graphs, approximating the graph convolution operation through a first-order Chebyshev polynomial. Supposing a graph *G* with *N* nodes, each node possessing its own features, let these node features form a matrix *X* ∈ *R*^*N*×*D*^. With an input feature matrix *X* and an adjacency matrix *A*, we can obtain the output:


(8) $$\begin{array}{l} Y=\sigma \left({\hat{D}}^{-\frac{1}{2}}A{\hat{D}}^{-\frac{1}{2}}XW\right) \end{array}$$

Where σ represents the nonlinear activation function.

### 3.2 Channel attention mechanism based on average pooling and max pooling

**Figure 3:**
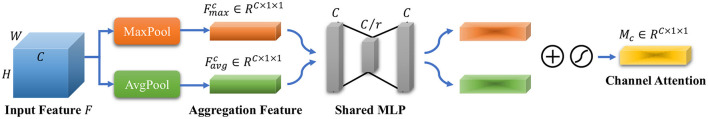
Schematic diagram of AM-CAM. As illustrated, the channel attention sub-module utilizes both the max pooling output and average pooling output with a shared network.

Firstly, we employ an autoencoder layer to perform re-representation of the input data, creating inputs with richer semantic information, as depicted in Figure 2, where the input channels are 30 and the output channels are 128. Then, in order to focus the model on crucial parts of the input related to the fatigue detection category, we generate channel attention maps by exploiting inter-channel relationships of features. This is achieved through the design of a channel attention mechanism based on average pooling and max pooling (AM-CAM) layer. The channel attention mechanism focuses on determining “what” in the input is meaningful, treating each channel of the feature map as a feature detector (Zeiler and Fergus, 2014). To compute channel attention effectively, we compress the spatial dimensions of the input feature maps. To gather spatial information, we employ an average pooling layer to gain insights into the extent of the target object effectively, utilizing it in the attention module to compute spatial statistics. Additionally, we use a max pooling layer to collect salient information about different object features, enabling the inference of finer channel attention. Figure 3 illustrates the computation process of channel attention maps, and the detailed operations are described as follows.

Given an intermediate feature map *F* ∈ *R*^*C*×*H*×*W*^ as input, we first utilize average pooling and max pooling operations to aggregate spatial information from the feature map, generating two distinct spatial context descriptors: $F_{avg}^{c}$ and $F_{max}^{c}$, representing average-pooled features and max-pooled features, respectively. Subsequently, both of these descriptors are fed into a multilayer perceptron (MLP) with a hidden layer to generate the channel attention map $M_{c}\in R^{C\times 1\times 1}$. To reduce parameter overhead, the hidden activation size is set to $R^{\frac{C}{r}\times 1\times 1}$, where *r* is the reduction ratio and is set to 16 in our study. After applying the shared network to each descriptor, we merge the output feature vectors using element-wise summation. In short, the channel attention is computed as:


(9) $$\begin{array}{l} \begin{array}{c} M_{c}\left(F\right)=\sigma \left(MLP\left(AvgPool\left(F\right)\right)+MLP\left(MaxPool\left(F\right)\right)\right)\\ =\sigma \left(W_{1}\left(W_{0}\left(F_{avg}^{c}\right)\right)+W_{1}\left(W_{0}\left(F_{max}^{c}\right)\right)\right) \end{array} \end{array}$$

Where σ denotes sigmoid function, $W_{0}\in R^{\frac{C}{r}\times C}$ and $W_{1}\in R^{C\times \frac{C}{r}}$, Note that the MLP weights, *W*_0_ and *W*_1_, are shared for both inputs and the ReLU activation function is followed by *W*_0_. The output *F*_*out*_ of AM-CAM can be formulated as:


(10) $$\begin{array}{l} F_{out}=M_{c}\left(F\right)⊙F \end{array}$$

### 3.3 Multi-semantic dynamical graph convolution

In this study, we propose a multi-semantic dynamical graph convolution (MD-GC) for extracting spatial features from the input. It determines the adjacency matrix based on spatial adjacency relationships, Euclidean spatial distance, and self-attention mechanism. Our approach constructs data-driven intrinsic topology under various semantic patterns, enhancing the spatial feature extraction capability of graph convolution. Overall, given an intermediate feature map *X* ∈ *R*^*C*×*V*^ as input, the output of MD-GC can be computed as:


(11) $$\begin{array}{l} MDGC(X)\;=\;\sigma (BN(SRGC(X)\;+\;EDGC(X)\\ \;+\;SAGC(X))) \end{array}$$

Where σ is sigmoid function, *BN* is batch normalization, SRGC represents spatial relationship-based graph convolution, EDGC represents Euclidean distance-based graph convolution, and SAGC stands for self-attention-based graph convolution.

#### 3.3.1 Graph convolution based on spatial relationship

**Figure 4:**
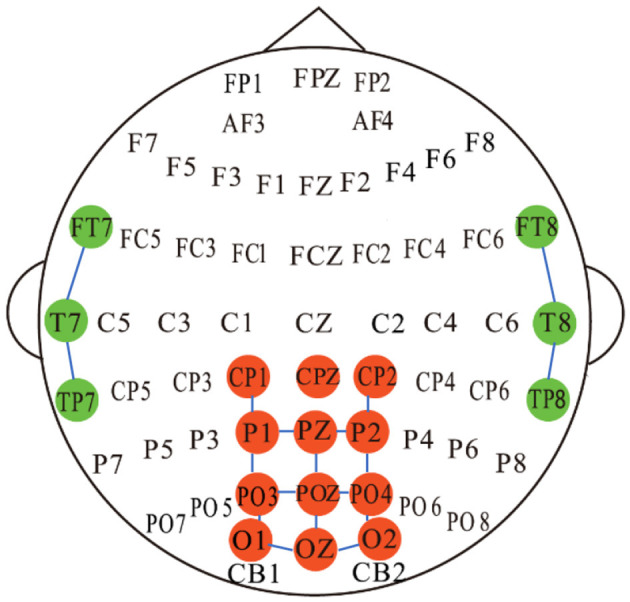
A schematic diagram illustrating the connections between the 17 EEG channels based on spatial adjacency relationships is used to construct the adjacency matrix for SRGC. CPZ serves as the reference electrode and is not involved in the construction of the adjacency matrix.

Intuitively, the correlation between EEG electrodes is constrained due to the distribution of nodes on the brain (Song et al., 2020), which represents inherent connections. To capture this relationship, we developed a spatial adjacency graph, denoted as *G*_*SR*_(*V, A*_*SR*_). *A*_*SR*_ represents the spatial adjacency matrix between brain nodes, as shown in Figure 4, where adjacent nodes are connected by solid blue lines. *A*_*SR*_ considers the adjacency relationships of 6 channels from the temporal region of the brain and 12 channels from the posterior part of the brain. We first normalize the spatial adjacency matrix *A*_*SR*_ using


(12) $$\begin{array}{l} {Ã}_{SR}=D_{SR}^{-1}A_{SR} \end{array}$$

$D_{SR}^{-1}\in R^{N\times N}$ is a diagonal degree matrix of *A*_*SR*_. Ã_*SR*_ provides nice initialization to learn the edge weights and avoids multiplication explosion (Brin and Page, 1998; Chen et al., 2018). Given the computed Ã_*SR*_, we propose the spatial relationship-based graph convolution (SRGC) operator. Let *X* ∈ *R*^*V*×*C*^ and $Y_{SRGC}\in R^{V\times C_{out}}$ be the input and output features of SRGC, respectively. The SRGC operator can be formalized as:


(13) $$\begin{array}{l} Y_{SRGC}=SRGC\left(X\right)={Ã}_{SR}XW_{SR}^{T} \end{array}$$

Where $W_{SR}\in R^{C_{out}\times C}$ is the trainable weight used to facilitate feature updating in the SRGC.

#### 3.3.2 Graph convolution based on Euclidean-space distance

Considering that SRGC can only capture relationships between nodes connected by physiological connections, here we introduce a Euclidean distance-based graph convolution (EDGC) operator to capture potential relationships between physically non-connected nodes, thereby imposing higher-order positional information. Specifically, we define a Euclidean space distance adjacency matrix for the potential sample dependencies in EDGC, where the adjacency weight between nodes *i* and *j* is calculated as:


(14) $$\begin{array}{l} a_{i,j}=max\left(E\right)-e_{i,j} \end{array}$$

where *e*_*i,j*_ is an element at row *i* and column *j* in the matrix *E* ∈ *R*^*V*×*V*^ that represents the distance between every pair of nodes. To calculate *e*_*i,j*_, we first assume the input takes the form of *X* ∈ *R*^*V*×*C*^. Then, we have $e_{i,j}=∥{\bar{x}}_{i}-{\bar{x}}_{j}{∥}_{2}$, where $∥{\bar{x}}_{i}-{\bar{x}}_{j}{∥}_{2}$ represents the Euclidean spatial distance between nodes *i* and *j* in *X*. Finally, subtracting *e*_*i,j*_ from the maximum value in matrix *E* defines the adjacency relationship between nodes *i* and *j*, implying that nodes closer together have higher adjacency weights. Let $Y_{EDGC}\in R^{V\times C_{out}}$ be the output features of EDGC, the EDGC operator can be formulated as:


(15) $$\begin{array}{l} Y_{EDGC}=EDGC\left(X\right)=A_{ED}XW_{ED}^{T} \end{array}$$

Where $W_{ED}\in R^{C_{out}\times C}$ is the trainable weight used to facilitate feature updating in the EDGC.

#### 3.3.3 Graph convolution based on self-attention mechanism

In addition to EDGC, we also propose a novel module based on the self-attention mechanism for graph convolution (SAGC) to derive context-dependent intrinsic topology. Specifically, SAGC employs self-attention (Vaswani et al., 2017) on node features to infer intrinsic topology and uses topology as neighborhood vertex information for graph convolutions. A self-attention is an attention mechanism that relates different brain nodes. Considering all possible node relations, SAGC infers positive bounded weights, termed self-attention map, to represent the strength of relationships. For a given SAGC input *X* ∈ *R*^*V*×*C*^, we linearly project node representations *X* to the query and key of *D* dimensions with learnable matrices $W_{O},W_{K}\in R^{C\times D}$ to obtain a self-attention map, as shown in Eq.16.


(16) $$\begin{array}{l} A_{SA}=softmax\left(\frac{XW_{K}{\left(XW_{Q}\right)}^{T}}{\sqrt{D}}\right) \end{array}$$

Where *softmax* is used to normalize the self-attention map, *D* is the output channel size and $D=\frac{C}{8}$. The scaling factor $\frac{1}{\sqrt{D}}$ is used to ensure even distribution of data and avoid elements with large values in the self-attention map having small gradients during backpropagation, which could hinder the training of neural network. Then, let $Y_{EDGC}\in R^{V\times C_{out}}$ be the output features of SAGC, the SAGC operator can be formalized as:


(17) $$\begin{array}{l} Y_{SAGC}=SAGC\left(X\right)=A_{SA}XW_{SA}^{T} \end{array}$$

Where $W_{SA}\in R^{C_{out}\times C}$ is the trainable weight used to facilitate feature updating in the SAGC.

### 3.4 Spatial attention mechanism based on average pooling and max pooling

**Figure 5:**
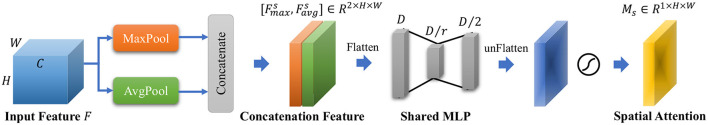
Schematic diagram of AM-SAM. As illustrated, the spatial attention sub-module utilizes both the max pooling output and average pooling output with a shared network.

After extracting spatial features, to retain crucial spatial node information and eliminate redundancy, we generate a spatial attention map based on the inter-spatial relationships between features. We design a spatial attention mechanism based on average pooling and max pooling (AM-SAM) to achieve this. Different from the channel attention, the spatial attention focuses on “where” is an informative part, which is complementary to the channel attention. Given an intermediate feature map *F* ∈ *R*^*C*×*H*×*W*^ as input, to compute the spatial attention map, we first apply average pooling and max pooling operations along the channel axis of *F* and concatenate them to generate an efficient feature descriptor. On the concatenated feature descriptors, we apply a multilayer perceptron (MLP) to generate the spatial attention map, which encodes emphasis or suppression of locations. The schematic diagram of AM-SAM is illustrated in Figure 5, and the detailed operational description of AM-SAM is as follows.

We aggregate channel information of a feature map by using two pooling operations, generating two 2D maps: $F_{max}^{s}\in R^{1\times H\times W}$ and $F_{avg}^{s}\in R^{1\times H\times W}$, which denotes average-pooled features and max-pooled features across the channel respectively. $F_{max}^{s}$ and $F_{avg}^{s}$ are first concatenated and flattened into $F_{fla}^{s}\in R^{2HW\times 1\times 1}$, which is then passed through a multilayer perceptron (MLP) with a hidden layer. To reduce computational resource consumption, the hidden layer size is set to $\frac{D}{r}$, where *D* = 2 × *H* × *W* and *r* is a reduction factor, set to 4 in our study. After obtaining the MLP's output, we use unflatten and nonlinear activation operation to transform the output into a two-dimensional spatial attention map. In short, the spatial attention is calculated as:


(18) $$\begin{array}{l} \begin{array}{c} M_{s}\left(F\right)=\sigma \left(MLP\left(\left[MaxPool\left(F\right);AvgPool\left(F\right)\right]\right)\right)\\ =\sigma \left(W_{1}\left(ReLU\left(W_{0}\left(\left[MaxPool\left(F\right);AvgPool\left(F\right)\right]\right)\right)\right)\right) \end{array} \end{array}$$

Where [·] denotes concatenation operation, σ is sigmoid function, $W_{0}\in R^{\frac{D}{r}\times D}$ and $W_{1}\in R^{\frac{D}{2}\times \frac{D}{r}}$. It is worth noting that [·] and *W*_1_ are followed by flatten and unflatten operations, respectively. The output *F*_*out*_ of AM-SAM can be formulated as:


(19) $$\begin{array}{l} F_{out}=M_{s}\left(F\right)⊙F \end{array}$$

## 4 Experiment

### 4.1 Method comparison

**Figure 6:**
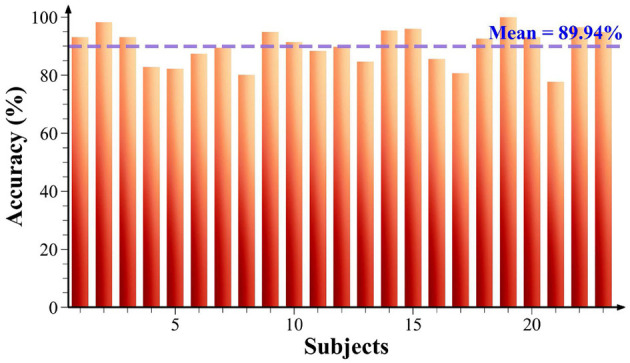
Fatigue detection accuracy of 23 subjects in the SEED-VIG dataset.

To better demonstrate the advancement of the AMD-GCN model, we compared it with the state-of-the-art methods on the SEED-VID dataset. Since the codes for these models was not publicly available, we followed the descriptions provided in the original papers for replication, so the final test results might differ. Here, PSD, DE, and WPCA represent different types of features extracted from the raw EEG signals. For the KNN classifier, we set the number of neighbors to 3. The SVM classifier utilized a radial basis function (RBF) kernel for training. EEGNet (Lawhern et al., 2018) is a single CNN architecture capable of accurately classifying EEG signals from various brain-machine interface paradigms. ESTCNN (Gao et al., 2019) is a spatio-temporal CNN model that emphasizes the temporal dependencies of each electrode and enhances the ability to extract spatial information from EEG signals. SAT-IFDM (Hwang et al., 2021) is a subject-independent model for classifying driver fatigue states, aimed at mitigating individual differences among subjects. LPCCs + R-SCM (Chen et al., 2022) is a novel psychological fatigue detection algorithm based on multi-domain feature extraction and fusion. It employs linear prediction to fit the current value with a set of past samples to calculate linear predictive cepstral coefficients (LPCCs) as temporal features. PDC-GCN (Zhang et al., 2020) has been introduced in the section slowromancapi@. GCNN-LSTM (Yin et al., 2021) is a model that combines GCN and LSTM. The model uses GCN for feature extraction and processes the obtained features using LSTM, followed by classification using dense layers. The chosen models for comparison are relatively representative and reproducible. Figure 6 presents the fatigue detection accuracy of all subjects using the AMD-GCN model on the SEED-VIG dataset, and the results of model comparisons are reported in Table 2.

**Table 2:** Comparison with accuracy and individual variation of state-of-the-art methods on the SEED-VIG dataset.

**Method**	**Accuracy (%)**	**IV (Individual variation)**
DE-KNN	77.37	15.45
PSD-SVM (Barua et al., 2019)	77.64	20.41
DE-SVM (Barua et al., 2019)	78.60	19.10
WPCA-SVM (Dong et al., 2019)	79.71	17.69
EEGNet (Lawhern et al., 2018)	84.50	13.24
ESTCNN (Gao et al., 2019)	86.55	11.23
SAT-IFDM (Hwang et al., 2021)	85.28	11.50
LPCCs + R-SCM (Chen et al., 2022)	87.10	8.07
PDC-GCN (Zhang et al., 2020)	89.42	10.22
GCNN-LSTM (Yin et al., 2021)	89.31	10.45
AMD-GCN (Ours)	**89.94**	**6.14**

The bold values represent the best accuracy and individual variation.

Obviously, Figure 6 shows that the detection accuracy is 77.74% for 21-th subject, while the detection accuracy for the remaining participants is all above 80%, and even 19-th subject achieved 100% accuracy. This indicates that the AMD-GCN model possesses great generalization capabilities and has the potential to achieve fatigue detection for a wide range of drivers. As can be seen in Table 2, our proposed AMD-GCN model has an accuracy improvement of about 10.23~12.57% compared to the traditional machine learning methods (KNN, SVM). Compared to CNN-based methods, the accuracy improvement is about 2.84~5.44%. Compared with the GCN-based method, the accuracy improvement is about 0.52~0.63%. The experimental results prove that the performance of the AMD-GCN model outperforms existing detection methods.

### 4.2 Ablation study

In this section, to further validate the impact of fused features and the role of each module in AMD-GCN, we performed a series of ablation studies, and the experimental results are documented in Table 3. From rows 2, 3, 10 of Table 3, it can be observed that the detection accuracy decreases by 2.75% and 3.66% when SEED-VIG-5band or SEED-VIG-2Hz is removed from the fused features, respectively. This indicates that both SEED-VIG-5band and SEED-VIG-2Hz are indispensable for enhancing the performance of EEG-based driver fatigue detection, and their effects are complementary. Furthermore, the detection accuracy of SEED-VIG-2Hz is higher by 0.91% compared to SEED-VIG-5band, indicating that DE features extracted from 25 frequency bands can better capture the heterogeneity of different fatigue states.

**Table 3:** Experimental results of ablation study on the SEED-VIG dataset, where w/o indicates the removal of specific functional module.

**Method**	**Accuracy (%)**	**IV (Individual variation)**
w/o SEED-VIG-5band	87.19^↓2.75^	7.06^↑0.92^
w/o SEED-VIG-2Hz	86.28^↓3.66^	7.61^↑1.47^
w/o AM-CAM	86.47^↓3.47^	7.56^↑1.42^
w/o MD-GC	82.64^↓7.30^	9.44^↑3.30^
w/o AM-SAM	87.98^↓1.96^	6.81^↑0.67^
w/o SRGC	88.03^↓1.91^	6.75^↑0.61^
w/o EDGC	86.65^↓3.29^	7.33^↑1.19^
w/o SAGC	85.92^↓4.02^	7.89^↑1.75^
AMD-GCN	**89.94**	**6.14**

The down and up arrow indicates a decrease in accuracy and an increase in individual variation after the removal of specific functional modules, respectively. The bold values represent the best accuracy and individual variation.

Rows 4, 5, and 6 of Table 3 shows the detection accuracy of the AMD-GCN without the AM-CAM, MD-GC, and AM-SAM functional modules, respectively. Firstly, the AM-CAM module is beneficial to aid the model in focusing on important information related to fatigue detection, and removing the AM-CAM module could introduce noise and confusion to fatigue state detection. The experimental results indicate that AM-CAM contributes to a 3.47% accuracy improvement for the model. Secondly, MD-GC can establish adjacency topologies of numerous semantic patterns, enabling rich non-Euclidean spatial feature learning. Removing MD-GC would disregard functional connections and inherent relationships between EEG nodes, thus weakening the performance of AMD-GCN and reducing the model accuracy by 7.3%. Furthermore, the AM-SAM module can eliminate redundant spatial node information from the output of MD-GC, aiding in enhancing the network's capability to differentiate data from different fatigue states. The experimental results show that AM-SAM contributes to a 1.96% accuracy improvement for the model. In summary, the designed modules successfully enhance the performance of EEG-based driving fatigue detection.

To validate the effectiveness of the adjacency topologies for the three semantic patterns in MD-GC, we obtained the detection accuracy of AMD-GCN without SRGC, EDGC, and SAGC, as described in rows 7, 8, and 9 of Table 3. Apparently, AMD-GCN without SRGC, EDGC, SAGC achieve 88.03%, 86.65%, 85.92%, underperforming the vanilla one by 1.91%, 3.29%, 4.02% respectively. The intrinsic topologies of these semantic patterns are crucial for AMD-GCN to learn category-dependent and data-dependent spatial features, which enhance the performance of AMD-GCN significantly. Moreover, it is evident that the improvements brought by these graph convolutions based on different semantic patterns can be superimposed, implying their roles are complementary to each other.

### 4.3 Supplement experiment

**Figure 7:**
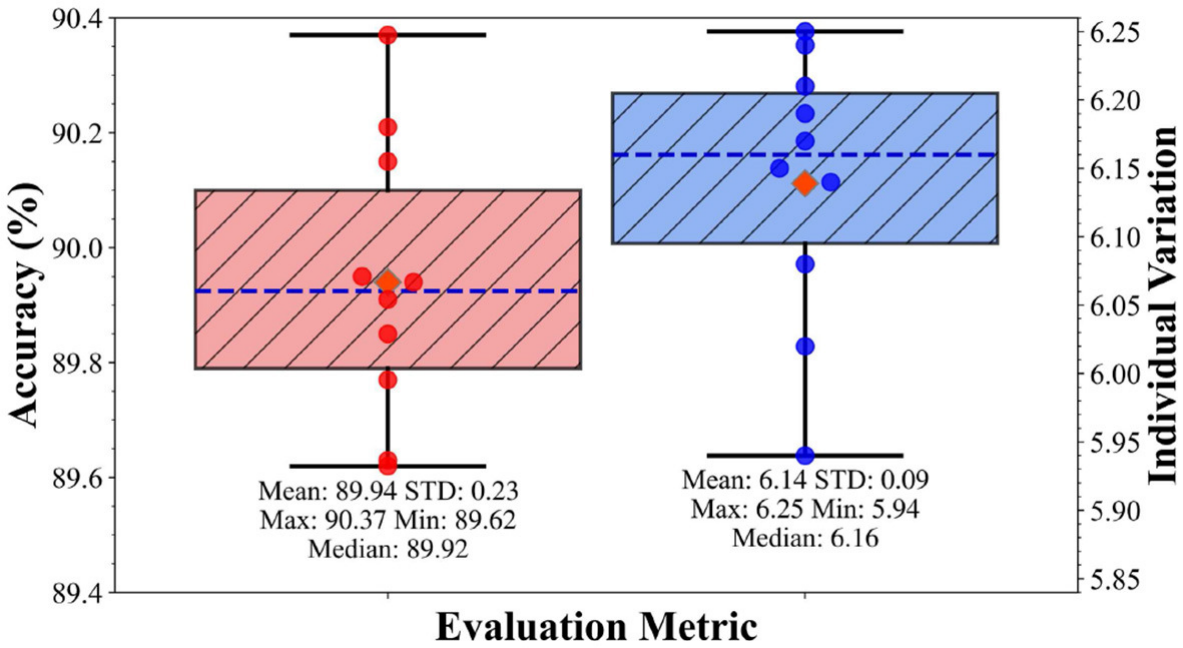
Results of 10 repeated experiments. The orange diamond points represent the mean value, the deep blue dashed lines represent the median value, the red and blue scattered points denote the accuracy and individual variations of the repeated experiments, respectively.

To verify the reliability of our algorithm, we conducted 10 repeated experiments on the SEED-VIG dataset. In each experiment, the dataset was randomly divided into 5 folds, with one fold used for testing and the remaining four for training, the results are depicted in Figure 7. It can be found that the accuracy varies from 89.62% to 90.37%, and individual variations range from 5.94 to 6.25, this indicates the stability of our method in terms of both detection accuracy and individual variation metrics. Figure 7 presents an average accuracy of 89.94% and an average individual variation of 6.14 for the AMD-GCN, both of which surpass the state-of-the-art methods reported in Table 2. Note that the values reported in Table 2 are average accuracy and average individual variation.

**Figure 8:**
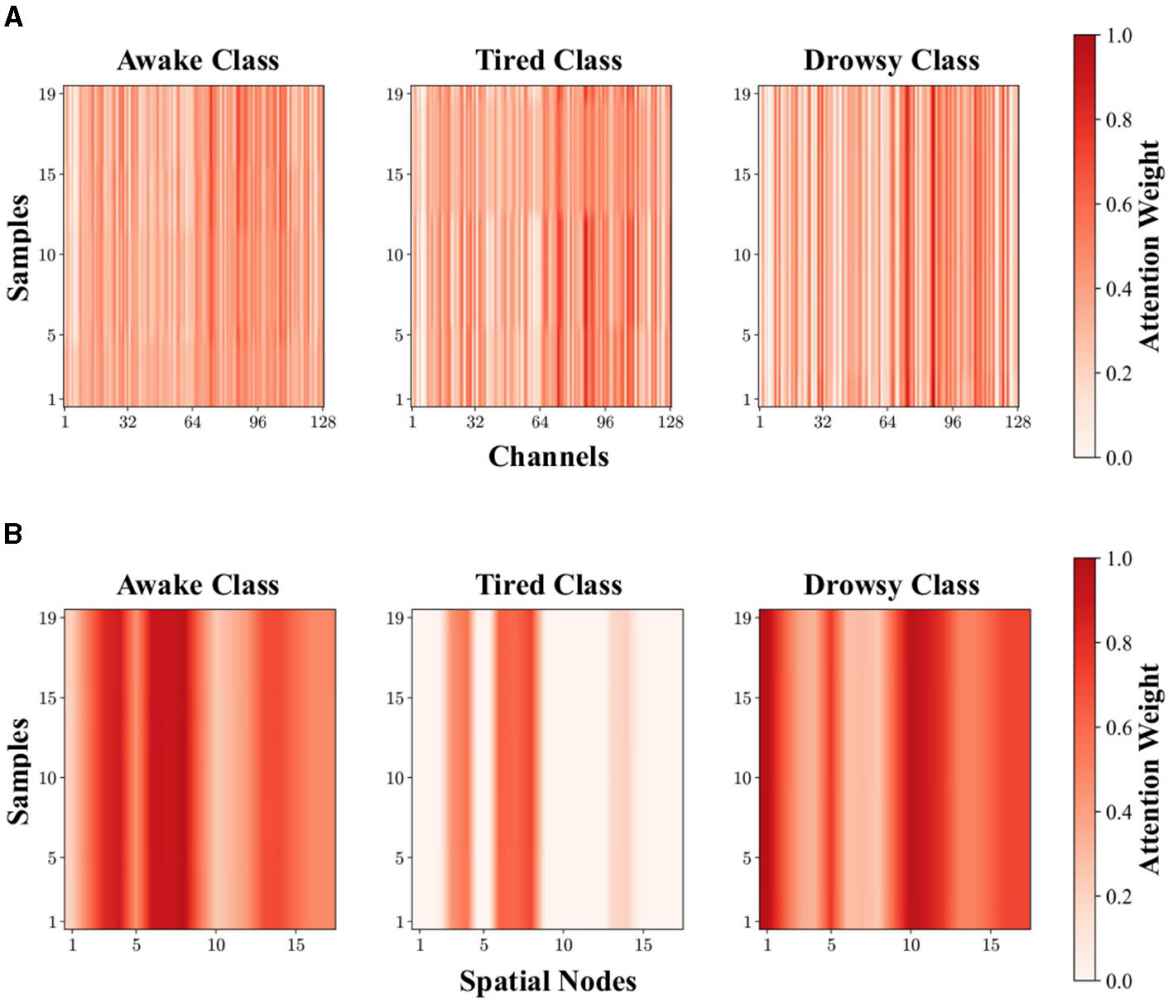
The visualization of attention map for the first subject under different fatigue states. **(A)** Channel attention map. **(B)** Spatial attention map of first layer.

Then, we visualize the channel attention map and spatial attention map of first layer for the first subject under three fatigue states, as shown in Figure 8. Obviously, AM-CAM can achieve channel filtering for inputs with richer semantic information, allowing the model to capture essential parts of the input related to fatigue detection category, and AM-SAM is able to retain crucial spatial node information associated with fatigue states to mitigate interference from redundant information. It can be summarized that our proposed AM-CAM and AM-SAM effectively enhance the feature representation ability of neural network on input data, thereby improving the performance of EEG-based fatigue detection task.

**Figure 9:**
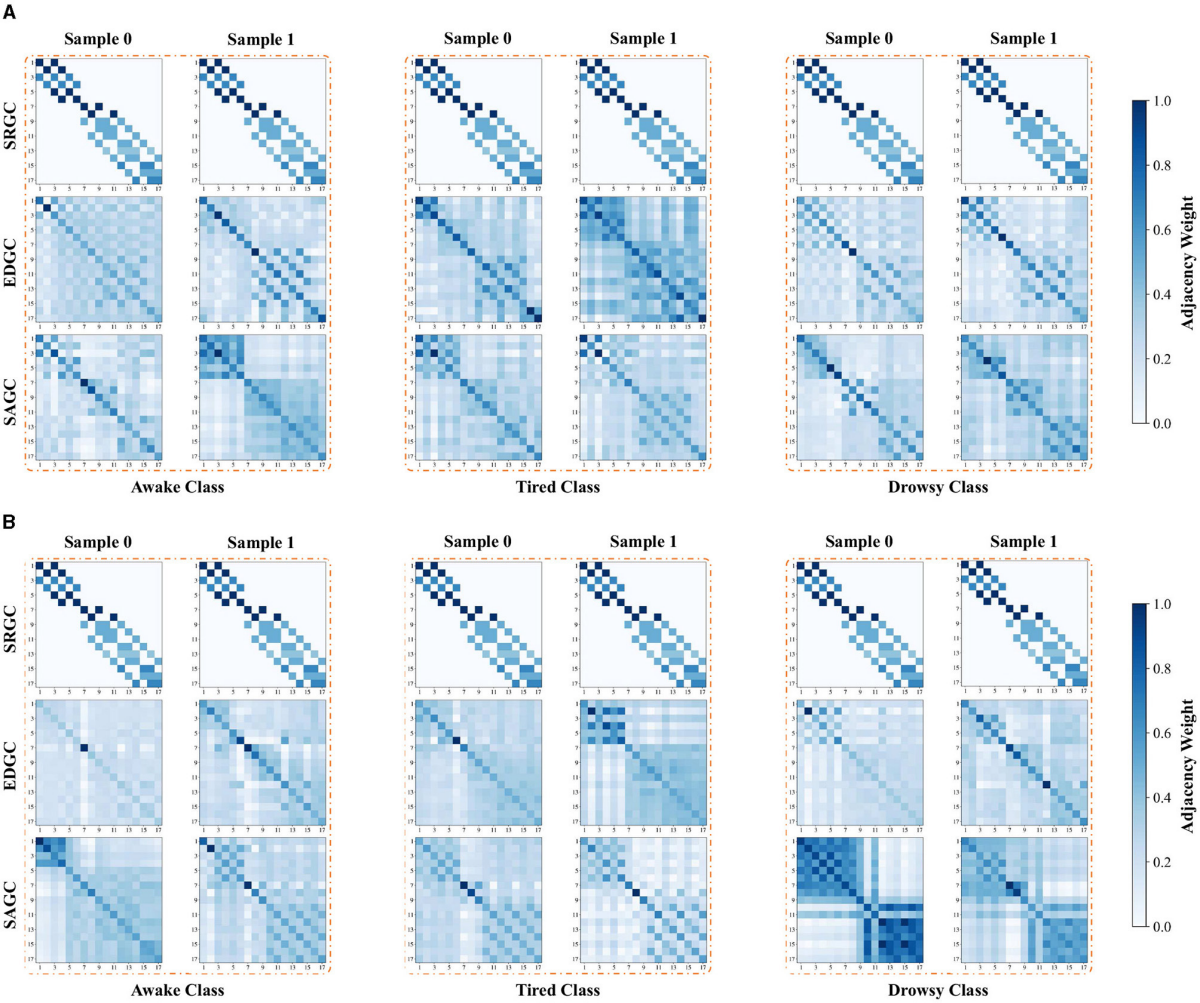
In the first layer of AMD-GCN, adjacency matrices for the three semantic patterns corresponding to samples of different fatigue states from two subjects. Sample 0 and Sample 1 represent two samples from the same category of the same subject. **(A)** First subject. **(B)** Second subject.

Furthermore, we visualize the adjacency matrices of the three semantic patterns constructed by AMD-GCN for different subjects, fatigue states, and samples, as shown in Figure 9. This can be concluded that due to SRGC containing a predetermined fixed adjacency graph, it remains consistent for all input data, thereby representing the inherent adjacency between brain nodes. In contrast, EDGC and SAGC construct intrinsic adjacency graphs based on the input data. They exhibit heterogeneity for different subjects, fatigue states, and samples, which benefits AMD-GCN in capturing potential data-dependent intrinsic adjacency relationships between brain nodes. This facilitates AMD-GCN in learning discriminative features for different fatigue states, thus enhancing the performance of driver fatigue detection. Additionally, from the adjacency matrices formed by SRGC, EDGC and SAGC, it can be observed that the adjacency weights among the 6 channels in the temporal region of the brain or the 11 channels in the posterior region of the brain are significantly stronger than the adjacency weights between the temporal and posterior regions. This consistency aligns with the brain tissue structure. Creating suitable adjacency matrices specifically for the temporal and posterior brain regions is crucial for efficient driver fatigue detection.

## 5 Conclusion

In this work, we have designed a driving fatigue detection neural network, referred to as the attention-based multi-semantic dynamical graph convolutional network (AMD-GCN), which integrates a channel attention mechanism, a spatial attention mechanism and a graph convolutional network. It aims to classify fused features extracted from EEG signals, where the fused features are obtained by concatenating DE features extracted from 5 frequency bands and DE features extracted from 25 frequency bands. In simple terms, we designed a channel attention mechanism based on average pooling and max pooling (AM-CAM), the mechanism helps the network retain crucial features in the input data that are relevant to driving fatigue detection. We introduced a multi-semantic dynamical graph convolution (MD-GC) that constructs intrinsic adjacency matrices for numerous semantic patterns based on input data., this enhancement improves the GCN's ability to learn non-Euclidean spatial features. We established a spatial attention mechanism (AM-SAM) based on average pooling and max pooling, enabling the network to eliminate redundant spatial node information from MD-GC outputs. Ultimately, we evaluated the performance of AMD-GCN on the SEED-VIG dataset, and the experimental results demonstrated the superiority of our algorithm, outperforming state-of-the-art methods in driving fatigue detection.

The limitations of the proposed AMD-GCN model are summarized from two aspects.

1) Although AMD-GCN model showed superior performance over existing deep learning models on the SEED-VIG dataset, its network architecture is still a shallow one which limits its feature learning ability in characterizing the underlying properties of EEG data.
2) We find significant differences in the recognition results of different subjects, indicating the existence of individual differences in the driving fatigue detection task. This has not yet been considered by AMD-GCN.
3) The outstanding performance of AMD-GCN is only evident in the subject-dependent experiments, but its performance has not been assessed in the subject-independent experiments.

As our future work, first, we intend to extend AMD-GCN into a deeper architecture to further enhance its data representation learning capacity. Second, we will investigate knowledge transfer strategies to mitigate cross-subject discrepancies in EEG-based driving fatigue detection. Third, we will utilize the leave-one-subject-out cross-validation strategy to evaluate the performance of AMD-GCN in subject-independent experiments on the large-scale fatigue detection dataset. Moreover, we plan to collect EEG fatigue data from numerous subjects and generate simulated volume conduction effect data for each subject, which aims to construct a novel fatigue detection dataset, to examine whether the learning process of the adjacency matrix by AMD-GCN from the raw EEG signals is influenced by spurious correlations introduced by volume conduction effects. We will also apply AMD-GCN to other physiological signals and adopt a combination of multiple physiological signals to comprehensively assess the driver's fatigue state.

## Data availability statement

Publicly available datasets were analyzed in this study. This data can be found here: https://bcmi.sjtu.edu.cn/home/seed/seed-vig.html.

## Author contributions

HL: Conceptualization, Formal analysis, Investigation, Methodology, Software, Writing—original draft. QL: Data curation, Formal analysis, Investigation, Methodology, Writing—original draft. MC: Conceptualization, Funding acquisition, Investigation, Resources, Supervision, Writing—review & editing. KC: Data curation, Investigation, Visualization, Writing—review & editing. LM: Software, Validation, Writing—review & editing. WM: Data curation, Formal analysis, Resources, Writing—review & editing. ZZ: Conceptualization, Data curation, Validation, Writing—review & editing. QA: Data curation, Validation, Visualization, Writing—review & editing.

## References

[B1] Abidi A. Ben Khalifa K. Ben Cheikh R. Valderrama Sakuyama C. A. Bedoui M. H. (2022). Automatic detection of drowsiness in EEG records based on machine learning approaches. Neural Process. Lett. 1–25. 10.1007/s11063-022-10858-x23972332

[B2] Barua S. Ahmed M. U. Ahlström C. S. (2019). Automatic driver sleepiness detection using EEG, EOG and contextual information. Expert Syst. Appl. 115, 121–135. 10.1016/j.eswa.2018.07.054

[B3] Brin S. Page L. (1998). The anatomy of a large-scale hypertextual web search engine, in Proceedings of the 10th international conference on World Wide Web, 107–117, 10.1016/S0169-7552(98)00110-X

[B4] Cai Q. Gao Z. An J. Gao S. Grebogi C. (2020). A graph-temporal fused dual-input convolutional neural network for detecting sleep stages from EEG signals. IEEE Trans. Circuits Syst. II, 68, 777–781. 10.1109/TCSII.2020.3014514

[B5] Chen K. Liu Z. Liu Q. Ai Q. Ma L. (2022). EEG-based mental fatigue detection using linear prediction cepstral coefficients and Riemann spatial covariance matrix. J. Neural Eng. 19, 066021. 10.1088/1741-2552/aca1e2 36356315

[B6] Chen S. Tian D. Feng C. Vetro A. Kovacěvić J. (2018). Fast resampling of three-dimensional point clouds via graphs. IEEE Trans. Signal Process., 66, 666–681. 10.1109/TSP.2017.2771730

[B7] Chen W. Wang W. Wang K. Li Z. Li H. Liu S.. (2020). Lane departure warning systems and lane line detection methods based on image processing and semantic segmentation: a review. J. Traffic Transp. Eng. 7, 748–774. 10.1016/j.jtte.2020.10.002

[B8] Dinges D. F. Grace R. (1998). PERCLOS? a valid psychophysiological measure of alertness as assessed by psychomotor vigilance. US Department of Transportation, Federal Highway Administration, Publication Number FHWA-MCRT-98-006. US Department of Transportation; Federal Highway Administration.

[B9] Dong N. Li Y. Gao Z. Ip W. H. Yung K. L. A. (2019). WPCA-based method for detecting fatigue driving from EEG-based internet of vehicles system. IEEE Access, 7, 124702–124711. 10.1109/ACCESS.2019.2937914

[B10] Gao Z. Dang W. Wang X. Hong X. Hou L. Ma K.. (2021). Complex networks and deep learning for EEG signal analysis. Cogn. Neurodyn. 15, 369–388. 10.1007/s11571-020-09626-1 34040666 PMC8131466

[B11] Gao Z. Wang X. Yang Y. Mu C. Cai Q. Dang W.. (2019). EEG-based spatio-temporal convolutional neural network for driver fatigue evaluation. IEEE Trans. Neural Netw. Learn. Syst. 30, 2755–2763. 10.1109/TNNLS.2018.2886414 30640634

[B12] Huang R. Wang Y. Li Z. Lei Z. Xu M. (2022). Multi-granularity deep convolutional model based on feature recalibration and fusion for driver fatigue detection. IEEE Trans. Intell. Transp. Syst. 23, 630–640. 10.1109/TITS.2020.3017513

[B13] Hwang S. Park S. Kim D. Lee J. Byun H. (2021). Mitigating inter-subject brain signal variability for EEG-based driver fatigue state classification, in IEEE International Conference on Acoustics, Speech and Signal Processing, ICASSP. Toronto: IEEE, 990–994.

[B14] Jia H. Xiao Z. Ji P. (2023). End-to-end fatigue driving EEG signal detection model based on improved temporal-graph convolution network. Comp. Biol. Med. 152, 106431. 10.1016/j.compbiomed.2022.106431 36543007

[B15] Jia Z. Lin Y. Wang J. Ning X. He Y. Zhou R.. (2021). Multiview spatial-temporal graph convolutional networks with domain generalization for sleep stage classification. IEEE Trans. Neural Syst. Rehabil. Eng. 29, 1977–1986. 10.1109/TNSRE.2021.3110665 34487495 PMC8556658

[B16] Jia Z. Lin Y. Wang J. Zhou R. Ning X. He Y.. (2020). GraphSleepNet: Adaptive spatial-temporal graph convolutional networks for sleep stage classification. IJCAI. 1324–1330. 10.24963/ijcai.2020/184 PMC8556658 34487495

[B17] Kipf T. N. Welling M. (2016). Semi-supervised classification with graph convolutional networks. arXiv. 10.48550/arXiv.1609.02907

[B18] Ko W. Jeon E. Jeong S. Suk-I. H. (2021). Multi-scale neural network for EEG representation learning in BCI. IEEE Comput. Intell. Mag. 16, 31–45. 10.1109/MCI.2021.3061875

[B19] Lal S. K. Craig A. (2001). A critical review of the psychophysiology of driver fatigue. Biol. Psychol. 55, 173–194. 10.1016/S0301-0511(00)00085-5 11240213

[B20] Lal S. K. Craig A. (2002). Driver fatigue: electroencephalography and psychological assessment. Psychophysiology 39, 313–321. 10.1017/S0048577201393095 12212650

[B21] Lawhern V. J. Solon A. J. Waytowich N. R. Gordon S. M. Hung C. P. Lance B. J.. (2018). EEGNet: a compact convolutional neural network for EEG-based brain-computer interfaces. J. Neural Eng. 15, 056013. 10.1088/1741-2552/aace8c 29932424

[B22] Li Z. Li S. E. Li R. Cheng B. Shi J. (2017). Online detection of driver fatigue using steering wheel angles for real driving conditions. Sensors 17, 3, 495. 10.3390/s17030495 28257094 PMC5375781

[B23] Lin B. Huang Y. Lin B. (2019). Design of smart EEG cap. Comp. Methods Prog. Biomed. 178, 41–46. 10.1016/j.cmpb.2019.06.009 31416561

[B24] Papadelis C. Kourtidou-Papadeli C. Bamidis P. D. Chouvarda I. Koufogiannis D. Bekiaris E.. (2006). Indicators of sleepiness in an ambulatory EEG study of night driving, in 2006 International Conference of the IEEE Engineering in Medicine and Biology Society. New York, NY: IEEE, 6201–6204. 10.1109/IEMBS.2006.259614 17946748

[B25] Paulo J. R. Pires G. Nunes U. J. (2021). Cross-subject zero calibration driver's drowsiness detection: Exploring spatiotemporal image encoding of EEG signals for convolutional neural network classification. IEEE Trans. Neural Syst. Rehabil. Eng. 29, 905–915. 10.1109/TNSRE.2021.3079505 33979288

[B26] Peng B. Zhang Y. Wang M. Chen J. Gao D. (2023). T-A-MFFNet: Multi-feature fusion network for EEG analysis and driving fatigue detection based on time domain network and attention network. Comp. Biol. Chem. 104, 107863. 10.1016/j.compbiolchem.2023.107863 37023639

[B27] Quddus A. Shahidi Zandi A. Prest L. Comeau F. J. (2021). Using long short term memory and convolutional neural networks for driver drowsiness detection. Accid. Anal. Prev. 156, 106107. 10.1016/j.aap.2021.106107 33848710

[B28] Saeb S. Lonini L. Jayaraman A. Mohr D. C. Kording K. P. (2017). The need to approximate the use-case in clinical machine learning. GigaScience 6:gix019. 10.1093/gigascience/gix019 28327985 PMC5441397

[B29] Shi J. Wang K. (2023). Fatigue driving detection method based on Time-Space-Frequency features of multimodal signals. Biomed. Signal Proc. Control. 84, 104744. 10.1016/j.bspc.2023.104744

[B30] Song T. Zheng W. Song P. Cui Z. (2020). EEG emotion recognition using dynamical graph convolutional neural networks. IEEE Trans. Affect. Comp. 11, 532–541. 10.1109/TAFFC.2018.2817622

[B31] Song X. Yan D. Zhao L. Yang L. (2022). LSDD-EEGNet: an efficient end-to-end framework for EEG-based depression detection. Biomed. Signal Process. Control, 75, 103612. 10.1016/j.bspc.2022.103612

[B32] Vaswani A. Shazeer N. Parmar N. Uszkoreit J. Jones L. Gomez A. N.. (2017). Attention is all you need, in Advances in Neural Information Processing Systems, 5998–6008.

[B33] WHO (2009). Global Status Report on Road Safety: Time for Action. Geneva: World Health Organization.

[B34] Wu E. Q. Deng P. Qiu X. Tang Z. Zhang W. Zhu L.. (2021). Detecting fatigue status of pilots based on deep learning network using EEG signals. IEEE Trans. Cogn. Dev. Syst. 13, 575–585. 10.1109/TCDS.2019.2963476

[B35] Wu Y. Ji Q. (2019). landmark detection: a literature survey. Int. J. Comput. Vis. 127, 115–142. 10.1007/s11263-018-1097-z

[B36] Yao C. L. Lu B. L. (2020). A robust approach to estimating vigilance from EEG with neural processes, in IEEE International Conference on Bioinformatics and Biomedicine, Seoal: IEEE, 1202–1205.

[B37] Yin Y. Zheng X. Hu B. Zhang Y. Cui X. (2021). EEG emotion recognition using fusion model of graph convolutional neural networks and LSTM. Appl. Soft Comput. 100, 106954. 10.1016/j.asoc.2020.106954

[B38] Zeiler M. D. Fergus R. (2014). Visualizing and understanding convolutional networks, in Procedings of European Conference on Computer Vision (ECCV) Computer Vision (ECCV).

[B39] Zeng H. Li X. Borghini G. Zhao Y. Aricò P. Di Flumeri G.. (2021). An EEG-based transfer learning method for cross-subject fatigue mental state prediction. Sensors 21, 2369. 10.3390/s21072369 33805522 PMC8036954

[B40] Zhang T. Wang X. Xu X. Chen C. P. (2019). GCB-Net: Graph convolutional broad network and its application in emotion recognition. IEEE Trans. Affect. Comput. 13, 379–388. 10.1109/TAFFC.2019.2937768

[B41] Zhang W. Wang F. Wu S. Xu Z. Ping J. Jiang Y.. (2020). Partial directed coherence based graph convolutional neural networks for driving fatigue detection. Rev. Sci. Instrum. 91, 074713. 10.1063/5.0008434 32752838

[B42] Zhang Y. Guo R. Peng Y. Kong W. Nie F. Lu L. B.. (2022). An auto-weighting incremental random vector functional link network for EEG-based driving fatigue detection. IEEE Trans. Instrument. Measure, 71, 1–14. 10.1109/TIM.2022.3216409

[B43] Zheng W. Lu L. B. (2017). A. multimodal approach to estimating vigilance using EEG and forehead EOG. J. Neural Eng. 14, 026017. 10.1088/1741-2552/aa5a98 28102833

[B44] Zhu J. Jiang C. Chen J. Lin X. Yu R. Li X.. (2022). based depression recognition using improved graph convolutional neural network. Comput. Biol. Med. 148, 105815. 10.1016/j.compbiomed.2022.105815 35917638

